# Menopause and Brain Health: Hormonal Changes Are Only Part of the Story

**DOI:** 10.3389/fneur.2020.562275

**Published:** 2020-09-23

**Authors:** Pauline M. Maki, Rebecca C. Thurston

**Affiliations:** ^1^Women's Mental Health Research Program, Department of Psychiatry, Psychology and Obstetrics and Gynecology, University of Illinois at Chicago, Chicago, IL, United States; ^2^Women's Biobehavioral Health Laboratory, Department of Psychiatry, Epidemiology, and Psychology, University of Pittsburgh, Pittsburgh, PA, United States

**Keywords:** menopause, cognition, vasomotor, brain, neuroimaging, cardiovascular

## Abstract

Most studies of menopause and brain aging have focused on the role of the sex steroid hormone, estradiol, as a key mechanisms contributing to cognitive and brain aging in women. An emerging literature demonstrates that beyond endogenous estradiol levels, menopausal symptoms, particularly vasomotor symptoms (VMS), are also key determinants of menopause-related changes in cognition and brain function. Critically, that literature shows the importance of using objective techniques to identify associations of VMS with memory performance, brain structure, and brain function. While self-report measures are important patient-centered outcomes in women's health research, objective measures of VMS typically relate more strongly to indices of cognitive and brain health. Currently, it is premature to make a causal claim about VMS and memory dysfunction, but initial findings raise the possibility that women with VMS might experience an improvement in cognition with VMS treatment. More generally, these findings underscore the utility of investigating female-specific risk factors for cognitive decline.

## Introduction

The burgeoning field of research in menopause and brain health has its roots in the discovery, now more than 30 years ago, by Catherine Woolley in the laboratory of Bruce McEwen that the structure and function of the hippocampus is influenced by changes in physiological levels of the sex steroid hormone, estradiol (1, 2). This foundational discovery propelled the growth of basic science research on the role of sex steroid hormones in brain function and brain structure, establishing a protective role in both rodent and non-human models. While there is a clear role for research on the role of sex steroid hormones in brain aging, comparatively little work has been conducted on the role of menopausal symptoms in brain aging. The hallmark symptom of the menopause is vasomotor symptoms (VMS), hot flashes and night sweats. These symptoms persist for many women well beyond the final menstrual period, into periods of risk for dementia, when levels of estradiol have plateaued (3). The continuity of VMS beyond the menopausal transition raises important questions about their potential role in cognition and brain health. This focus is important not only mechanistically but also clinically. If VMS contribute to cognitive dysfunction in women, any effective treatment for VMS regardless of whether the treatment is hormonal, non-hormonal, or lifestyle, could potentially confer cognitive benefit.

In this review, we present an overview of VMS in relation to cognition and brain function at midlife and beyond. We consider the methodologies used to measure VMS and the importance of using objective VMS measures in research studies. We draw on observational studies and clinical trials to show initial evidence of a relationship between VMS and memory dysfunction—the cognitive domain that appears most sensitive to female reproductive factors (4). We consider the mechanisms by which VMS can influence memory, drawing on our neuroimaging studies and on studies showing linkages to other risk factors for cognitive aging, particularly cardiovascular disease, and sleep dysfunction. We conclude that emerging evidence suggests a role for VMS in cognitive aging in women.

## VMS Epidemiology

VMS are the classic symptom of the menopause transition. VMS are experienced by most midlife women at some point during the menopause transition (5). For a third of women, VMS are frequent or severe. Whereas, VMS have long been assumed to be an incidental midlife symptom, more recent research has brought a wealth new knowledge about this prevalent midlife experience that has challenged long-held assumptions, elucidating their epidemiology and implications for women's health and functioning.

Whereas, VMS were once thought to be time-delimited events isolated to a few years around the onset on the postmenopause, recent data indicate that VMS persist for an average of 7–10 years for moderate-severe or frequent VMS, and much longer for milder symptoms (3, 6). Major cohort studies from the United States (US) and around the globe have further indicated that not all women follow the same trajectories of VMS over the transition (7, 8). For example, in the United States, 18% of women have VMS primarily when they are still cycling early in the transition, 29% of women have VMS have VMS primarily postmenopausally once their cycles have stopped, 27% of women have few or no VMS, and 25% of women have VMS that persist from early in the transition through the later postmenopausal years (7).

A growing body of research also underscores the pronounced racial/ethnic differences in VMS. In the United States, African American women have the most frequent, persistent, and bothersome VMS of any racial/ethnic groups, with over 80% of African American women reporting VMS at some point during the transition (9, 10). Approximately 50–70% of non-Hispanic White, Asian (Japanese, Chinese), and Hispanic report VMS during the menopause, with slightly (non-significantly) lower rates among Asian women. Similarly, in an Australian sample, Asian women have lower rates of VMS than other groups, and in an international consortium of menopause studies, Japanese women were less likely to report severe VMS than were Caucasian women (11, 12). Further, independent of race/ethnicity, less educated women and women in lower socioeconomic positions have more VMS than their more educated and affluent counterparts in both the United States (5) and in the United Kingdom (8).

VMS can have a profound impact on women's lives. They are a consistent predictor of depressed mood, sleep problems, and poorer quality of life during the menopause transition across social, emotional, and physical domains of functioning (13–15). In addition to these important implications for quality of life and functioning, recent data have also linked VMS to key indices of physical and neurocognitive health.

## VMS Physiology

Insights into the physiology of VMS has expanded greatly in recent years with the accumulation of clinical and basic science studies elucidating the critical role of hypothalamic KNDy (kisspeptin, neurokinin B and dynorphin) neurons (16). These neurons are located in the arcuate nucleus (infundibular nucleus in humans) of the hypothalamus, and play a critical role in the hypothalamic-pituitary-gonadal (HPG) axis. They are hypothesized to function as the gonadotropin releasing hormone (GnRH) pulse generator. In that role, KNDy neurons are thought to control the release of follicle stimulating hormone (FSH) and luteinizing hormone (LH), which stimulate ovarian production of estradiol. In turn, estradiol acts on KNDy neurons through negative feedback. As levels of estrogen decrease at menopause, KNDy neurons undergo hypertrophy (enlargement) and are reversed with estrogen supplementation (17). With respect to thermoregulatory function, KNDy neurons project to the median preoptic nucleus (MnPO) of the hypothalamus. There they bind to neurokinin 3 receptors (NK_3_R) which in turn project to heat dissipation effectors. The ligand for NK_3_R receptors is the endogenous neuropeptide, neurokinin B (NKB). As levels of estrogen decline, levels of NKB rise and activate NK_3_R receptors in the MnPO. This overactivation results in rapid heat dissipation response that women experience symptomatically as VMS. Targeting this mechanism, pharmacologic antagonists of NK_3_R are a new line of therapeutics to treat VMS in women, and lower VMS frequency and intensity more rapidly than conventional menopausal hormone therapy (HT) (18).

It has been known for quite some time that while declines in estradiol in the menopausal transition set the general stage for VMS and are therefore a necessary factor, changes in estradiol are not a sufficient proximal cause of individual VMS events (19). All women transition through the menopause but not all women have VMS. From a mechanistic perspective, the view that excessive NK3 signaling by NKB in the MnPO causes VMS accounts for the necessary role of estrogen withdrawal and for findings of a temporal, but not causal relationship of VMS and pulses of LH (16). However, other physiologic systems have been linked to VMS including the autonomic nervous system (particularly vagal withdrawal) (20, 21), the thermoregulatory system (22), and the hypothalamic pituitary adrenal axis (more fully elucidated below). Thus, significant advances have been made, yet further research is required to fully understand the underlying physiology of VMS, which is turn can advance the science on VMS and brain health.

## VMS Measurement: What Objective Techniques Tell Us That Self-Report Does Not

VMS are measured in several ways. Self-report approaches include questionnaires that ask women to recall their VMS from weeks, months, or years prior. In other work, diaries to report VMS are employed, and are completed at the end of the day, upon waking, or at the time of the VMS (23). However, these self-report measures have important limitations. It is well-established that the self-report of physical symptoms is subject to memory, mood, and other reporting processes (24–27). Important, VMS can also be assessed physiologically. The most well-established physiologic measure of VMS is sternal skin conductance (28). Early work supported the validity of this approach, which has been used in multiple subsequent observational studies and even clinical trials (29, 30). These physiologic measures are particularly important to understanding the etiology of the VMS and their relationships to key health indices apart from the influence of psychological, memory, and other reporting factors.

Psychological, sleep quality, and other non-VMS factors have a well-established impact on reporting of VMS. Typically, 62–72% of reported VMS are associated with an objective VMS, and 47–70% of objective VMS accompanied by a subjective report (31), with estimates varying by factors including whether the VMS occurred during sleep or wake, mood at the time of reporting, and the quality of the prior night's sleep. For example, more negative mood was associated with increased reporting of VMS not detected physiologically (31). Women with more negative mood also appraise their VMS to be more bothersome, apart from the occurrence of the VMS (32). Notably, physiologic VMS do not show placebo responses (in contrast to the 30% placebo response with diary-reported VMS (28, 30). Such evidence validates the physiologic VMS measures, as a “true” VMS measure that would not be expected to change with a placebo intervention. Further, physiologic VMS are particularly important when assessing nocturnal VMS. Accurately reporting VMS during sleep can be challenging and influenced by the quality of the sleep itself. For example, several studies with self-report and physiologic VMS measures show that reporting of VMS upon waking is influenced by the quality of sleep the prior night (27, 33). Some research indicates that reports of nighttime VMS recalled upon waking may be more related to sleep quality than to the occurrence of VMS themselves (33).

Finally, use of physiologic VMS measures is particularly important when investigating the links between VMS and cognition. Poorer memory performance may be associated with less accurate recall of VMS, thereby introducing a significant bias into the study when using self-reported VMS measures only. Despite this methodological limitation of self-report VMS, most of the literature relating VMS to memory performance relies on self-report measures of VMS. Ambulatory VMS monitors allow for real-time self-report of VMS events and therefore do not rely on recall for self-report VMS. In this way, studies using ambulatory monitors allow for the investigation of both self-report and physiologic VMS in relation to memory, without the influence of recall bias.

## VMS and Memory

In addition to VMS, cognitive complaints and objective cognitive difficulties are common in the menopausal transition. For example, among 12,425 healthy women 40–55 years of age in a baseline study of the SWAN, 39% of women complained of forgetfulness (34). Among those women, complaints of forgetfulness (yes/no) varied by menopausal status; compared to the premenopause, the odds of forgetfulness increased by 44% in the early perimenopause, 43% in the late perimenopause, 27% in the postmenopause, and 27% odds in surgical menopause (34). In the Seattle Midlife Women's Health Study, predictors of memory complaints included age, hot flashes, anxiety, depressed mood, perceived stress, perceived health and history of sexual abuse (35). Subjective memory complaints relate to objective cognitive difficulties in midlife women. In the Rochester Investigation of Cognition and Menopause (RICAM), memory complaints on a standardized questionnaire, the Memory Function Questionnaire, were associated with decreased encoding on a verbal memory test and with working memory (36) and in later work with working memory and attention/concentration (37). In our own work, subjective ratings of current memory were associated with performance on a measure of delayed verbal memory (38). Large-scale prospective studies of healthy women followed across the menopausal transition demonstrate subtle but reliable changes in verbal learning and memory, as well as processing speed (39, 40). Notably, initial evidence from these studies indicate that performance declines in the perimenopause but appears to normalize in the postmenopause (39, 40). If this pattern of change in memory across the menopausal transition is valid, it is difficult to envisage how declines in estradiol alone can account for changes in memory, as memory appears to bounce back while declines in estradiol persist.

A growing body of evidence suggests that menopausal symptoms, particularly VMS, may account in part for changes in memory in midlife women. Importantly, subjective VMS generally are unrelated to memory performance, including a study of 6-year longitudinal cohort study of 1,903 midlife women in SWAN (41). The studies showing evidence of a relationship between VMS and memory performance relied on the use of the ambulatory monitors (42–44). The first evidence emerged from a cross-sectional investigation of women with moderate-to-severe VMS (43). Women in that study wore ambulatory VMS monitors, recorded subjective VMS on that monitor and/or on a diary (useful for sleep VMS), and performed a battery of neuropsychological tests. A higher frequency of physiologic VMS but not subjective VMS, particularly during sleep, were associated with lower performance on a verbal memory test. Self-reported sleep, age, race, and mood did not account for those relationships. The association between VMS and worse memory was recently replicated in breast cancer survivors, again with associations evident only in physiologic VMS (42). In breast cancer survivors, effects were not differentially stronger during sleep. Importantly, these associations were independent of actigraphy-based measures of sleep, mood and other factors (42).

The findings from these cross-sectional studies of physiologic VMS and memory difficulties, raised the possibility that treating VMS may result in improvements in memory. To address that question, we examined changes in memory performance and physiological VMS in a pilot randomized, sham-control study of stellate ganglion blockade (SGB) (44). SGB is an anesthesia procedure used in pain medicine and involves administration of a local anesthetic to the C6 ganglion. Consistent with findings from open-label trials (45–48), SGB reduced moderate-to-severe VMS and reduced physiologic VMS compared to sham (30). While SGB did not significantly improve memory, the magnitude of improvement in physiologic VMS was significantly associated with the magnitude of improvement in memory performance (44). Those findings suggested a linear relationship between improvements in VMS frequency and improvements in memory, and raise the possibility that other hormonal and non-hormonal interventions for VMS may confer memory benefits.

## VMS and Brain Health

In a series of neuroimaging studies in midlife women, physiological VMS but not subjective VMS, were associated with brain health. In MsHeart, the frequency of physiologic VMS was positively associated with increases in white matter hyperintensities (49). White matter hyperintensities (WMH) are risk factors for AD and vascular dementia. Whether VMS are causally related to WMH is unknown, but the pathophysiological basis of this association is likely to involve cardiovascular disease (CVD) risk factors, as CVD risk factors are linked to both VMS and WMH (50).

In addition to brain structure, physiologic VMS are associated with alterations in brain function measured using functional magnetic resonance imaging (fMRI), including resting state functional connectivity (51) and blood-oxygen-level dependent (BOLD) fMRI during performance of a verbal memory task (52). In Ms. Heart, we investigated the relationship of VMS with alterations in the default mode network (DMN) during the resting state (52). The DMN is an organized network in the brain that is active during rest and suppressed during tasks requiring attention to the external world. The DMN includes regions along the anterior and posterior midline, the lateral parietal cortex, prefrontal cortex, and medial temporal lobe, including the hippocampus (53). In Ms. Heart, the frequency of VMS was associated with alterations in this network at rest as measured with fMRI (51). The pattern of alterations was characterized by hyperconnectivity of the hippocampus with multiple frontal regions, and this hyperconnectivity was especially pronounced for sleep VMS. Importantly, these associations persisted after controlling for age, race, education, and sleep. Hyperconnectivity in the DMN has been viewed as reflecting a fundamental response to neurological disruption (54), often viewed as compensatory in nature. As in the memory studies, physiologic VMS but not reported VMS were associated with alterations in the DMN, underscoring the importance of objective methods for VMS ascertainment.

In a recent fMRI study, we examined the association between VMS and brain activation during performance of a verbal memory task, including word encoding and word recognition conditions (52). Physiologic VMS but not reported VMS were associated with decreased memory and with alterations in brain activity during the memory task. During word encoding, more frequent physiologic VMS were positively associated with activation in the left orbitofrontal cortex, left middle frontal gyrus/superior frontal gyrus, right superior frontal gyrus, and right parahippocampus. During recognition, more frequent VMS were positively associated with activation in bilateral middle frontal and superior frontal regions and bilateral hippocampus/parahippocampus. Overall, these data suggest that VMS-related declines in memory may be due to alterations in the function of the hippocampus, parahippocampus, and multiple regions of the prefrontal cortex.

Together, the two fMRI studies present a consistent pattern that drives ongoing work in this area. The areas of the DMN associated with physiologic VMS—the hippocampus, medial frontal and orbitofrontal cortex—overlapped considerably with the brain areas associated with VMS in the memory study. In both cases, the patterns were of increased connectivity or activity, consistent with a compensatory response. Neither study found an association between brain function and subjective VMS.

## VMS and Brain Health Putative Mechanisms

### Cardiovascular

VMS have been linked to multiple indicators of CVD risk. In both cross-sectional and longitudinal studies, self-reported VMS have been associated with a more adverse CVD risk factor profile, including high blood pressure and hypertension risk, higher lipids (total cholesterol, LDL cholesterol, triglycerides, ApoB), a more insulin resistant profile, diabetes risk, and in some studies, a more pro-inflammatory or pro-coagulant profile (55). Controlling for traditional CVD risk factors, both self-reported. In the National Institutes of Health-funded MsHeart study, physiologically assessed presence or frequency of VMS have also been linked to poorer endothelial function, particularly for younger midlife women (56, 57). In MsHeart and other studies, more frequent self-reported and physiologically assessed VMS have been associated with greater carotid atherosclerosis (intima media thickness and plaque) (58–60). Other work suggests that reported VMS may also be associated with increased risk of future clinical CVD events (e.g., myocardial infarction, heart failure, stroke) and CVD mortality, which may depend on the timing of the VMS (61, 62). These associations between VMS and CVD risk generally are not explained by endogenous estradiol levels when these measurements are available. Notably, poorer cardiovascular health (e.g., adverse CVD risk factors, subclinical and clinical CVD) is a well-established risk factor for dementia (63–66). Thus, CVD risk should be considered as one mechanism linking VMS to poorer cognitive health and dementia risk.

### Cortisol and Blood Flow

VMS are associated with elevated levels of cortisol, a steroid hormone that is released by the adrenal glands in response to physical and psychological stress and activation of the hypothalamic-pituitary-adrenal (HPA) axis (67–69). Cortisol levels increase 20 min after a VMS, as measured objectively by changes in finger skin temperature and skin resistance (67). Increased cortisol may be one factor contributing to cognitive difficulties in midlife women with VMS. Elevated cortisol has been linked to decreases in memory and executive functioning, particularly in women (70–73). Levels of glucocorticoids at both high and low levels, in an inverted-U dose response curve, are associated with suboptimal performance on learning and memory retrieval tasks (74). Other work demonstrates that VMS frequently cause a reduction in blood flow to the brain (~5% reduction) (75).

### Sleep

In addition to VMS, sleep problems are common during the menopause transition, with up to half of midlife women reporting sleep problems (14). In longitudinal cohort studies, reported VMS are one of the most consistent predictors and robust of reported sleep problems during the menopause transition (76). Although findings employing objective measures of sleep and VMS initially produced somewhat mixed findings, several recent studies using objective measures of both VMS and sleep have further supported the importance of VMS to sleep (77). For example, we found a 5-fold odds of actigraphy-detected wakening with an objective VMS than during times without objective VMS; these wake episodes were observed irrespective of whether the woman reported the VMS or even knew she was having VMS (78). This poor sleep can have implications for women's brain health. We have found that women with greater objectively-detected wake after sleep onset had increased WMH even after considering multiple covariates including VMS (79). Sleep disturbance negatively affects cognitive function during healthy aging (80) and is associated with an increased risk of Alzheimer's disease (81). VMS may therefore negatively impact cognition and brain health through disrupted sleep quality.

## Discussion

An emerging body of work indicates that VMS may be an important determinant of cognition at midlife and beyond. This association emerged with the use of objective techniques for measuring VMS, and was not evident with self-report. About 70% of women will experience VMS and it is now recognized that this hallmark menopausal symptom persists in many women for more than a decade after the final menstrual period. While the focus on estradiol and brain health is clearly justified on the basis of a wealth of basic science studies, this emerging body of work supports the need to expand the focus beyond hormonal effects in menopause to menopausal symptoms. This focus is all the more important because of the possibility that VMS might be a modifiable risk factor for memory dysfunction.

Initial findings from a pilot study raise the possibility that women with VMS might experience an improvement in cognition once treated. It is clear that currently no strong claim can be made about a causal relationship between VMS and memory decline, but at minimum VMS may help to identify women at risk for memory decline at midlife.

At the systems level, our ongoing work in MsBrain funded by the National Institute on Aging explores relationships between physiologic VMS and brain health across a range of neuroimaging outcomes, including fMRI measures of verbal memory, resting state, and emotion perception; WMH; diffusion tensor imaging; and brain volume in a large sample of women who also contribute neuropsychological assessments, actigraphy-based measures of sleep, measures of subclinical CVD and CVD risk factors, hormone measures, and a wide range of sociodemographic and clinical variables. Findings from that study will not only provide insights into the reliability of initial findings in MsHeart, but will also allow for a more comprehensive assessment of the complex array of factors that link VMS to cognitive health. Ultimately, this line of research will contribute to a growing literature identifying female-specific risk factors for cognitive aging.

## Author Contributions

PM and RT each contributed to the writing of this review. All authors contributed to the article and approved the submitted version.

## Conflict of Interest

PM has served as a consultant to: Abbvie, Balchem, and Pfizer. RT has served as a consultant/advisor to Astellas, Pfizer, Procter & Gamble, and Virtue Health.
